# Chin wing osteotomy - a new facial concept

**DOI:** 10.1186/1746-160X-10-S1-O6

**Published:** 2014-12-12

**Authors:** Albino Triaca

**Affiliations:** 1 Oral and Maxillofacial Unit, Klinik Pyramide am See, Zürich, Switzerland

## 

A new technique for an extended genioplasty, the mandibular wing osteotomy, is presented step by step. This osteotomy allows correcting the position of the chin-prominence, to change the aspect of the divergence in the mandibular angulus and to achieve a lip competence in cases where it is needed. All these movements of the lower border of the mandibula can be performed completely independently from the positioning of the bases that is necessary for the correction of the malocclusion.

A planning concept is shown and the different treatment modalities for Angle class II and III malocclusions are presented.

